# Early History, Mealtime Environment, and Parental Views on Mealtime and Eating Behaviors among Children with ASD in Florida

**DOI:** 10.3390/nu10121867

**Published:** 2018-12-02

**Authors:** Heewon L. Gray, Sweta Sinha, Acadia W. Buro, Chantell Robinson, Karen Berkman, Heather Agazzi, Emily Shaffer-Hudkins

**Affiliations:** 1 College of Public Health, University of South Florida, 13201 Bruce B Downs Blvd, Tampa, FL 33612, USA; swetasinha@health.usf.edu (S.S.); acadia@health.usf.edu (A.W.B.); cnr@health.usf.edu (C.R.); 2 Center for Autism & Related Disabilities and Department of Child and Family Studies, University of South Florida, 13301 Bruce B Downs Blvd, Tampa, FL 33612, USA; kberkman@usf.edu; 3 Department of Pediatrics, University of South Florida, 13101 Bruce B Downs Blvd, Tampa, FL 33612, USA; hcurtiss@health.usf.edu (H.A.); eshaffer@health.usf.edu (E.S.-H.)

**Keywords:** autism spectrum disorder, mealtime behavior, children, diet, feeding behavior

## Abstract

This study was a cross-sectional study to examine problematic mealtime behaviors among children with autism spectrum disorder (ASD) in Florida. Forty-one parents completed a 48-item survey. The mean age of their children was 8.1 years and 73% were male. The data were divided and compared by age group: Ages 2–6, 7–11, and 12–17. Data from the 3- to 6-year-old children were extracted and compared with the references from Provost et al. (2010). There were age differences in eating difficulties at home (*p* = 0.013), fast food restaurants (*p* = 0.005), and at regular restaurants (*p* = 0.016). The total mealtime behavior score was significantly higher in early childhood (*p* < 0.001) and mid-childhood (*p* = 0.005) than adolescents. More parents of ages 3–6 with ASD reported difficulties with breastfeeding (*p* < 0.01); concerns about eating (*p* < 0.001); difficulties related to mealtime locations (*p* < 0.05), craving certain food (*p* < 0.05), and being picky eaters (*p* < 0.01) compared to typically developing children. The total mealtime behavior score was significantly higher in children with ASD than typically developing children (*p* < 0.001). The results indicate that early childhood interventions are warranted and further research in adolescents is needed.

## 1. Introduction

Children with autism spectrum disorder (ASD) frequently exhibit feeding difficulties and problematic mealtime behaviors [1,2,3] and consume fewer types of foods compared to typically developing children [4,5,6]. Food selectivity, or consuming a narrow variety of foods [7], has been associated with other problematic, ritualistic behaviors, which may contribute to inadequate nutrient intake [3,8,9,10]. A meta-analysis and review study indicated that children with ASD experience about five times more feeding problems and significantly lower intake of calcium and protein compared to a typically developing comparison group [2]. In addition, selective eating has been associated with preference for energy-dense foods such as sweetened beverages and snack foods as well as with inadequate consumption of nutrient dense foods such as fruit, vegetables, lean protein, and foods high in fiber [11]. Such imbalanced eating behaviors may contribute to the high prevalence of obesity in children with ASD. Children with ASD are up to 40% more likely to be obese than typically developing children [12,13,14]. In addition, adults with ASD are at high risk for developing hyperlipidemia, diabetes, coronary heart disease, and cancer [15]. Taken together, addressing problematic mealtime behaviors that may contribute to developing unhealthy dietary habits early in childhood is critical. Parents of children with ASD have also reported concerns regarding their children’s mealtime behaviors and the potential impact of these behaviors on their children’s nutritional status and well-being [9,16]. Furthermore, it has been reported that selective eating and problematic mealtime behaviors persist through the adolescent years [17,18]; however, whether or not there are any age differences in these behaviors has not been further examined. To better understand any demographic or regional differences in feeding or mealtime behaviors among children with ASD, further studies are needed with samples from diverse demographic backgrounds and regions.

The purpose of this study was to examine mealtime behaviors in children who are clinically diagnosed with ASD, specifically among children who reside in Florida. As some feeding difficulties can be recognized at a very early age, we selected a questionnaire that includes questions on children’s early feeding history. Provost et al. (2010) developed and validated a 48-item mealtime behavior survey to assess early history regarding feeding difficulties, mealtime location, problematic mealtime behaviors, and food preferences and restrictions [19]. Children and adolescents up to 17 years old were included in the current study, and to better understand any apparent age differences in problematic mealtime behaviors, we conducted comparison analyses among three sub-age groups (early childhood, mid-childhood, and adolescents). The objectives of this study were (1) to assess early history of feeding difficulties, mealtime location, problematic mealtime behaviors, and food preferences and restrictions of children with ASD in Florida; (2) to examine whether the results are different by age group; and (3) to compare the results from children aged 3 to 6 in our sample to reference data from Provost et al. [19]. The measurement was used to systematically compare the eating behaviors of young children with ASD (*n* = 24) to those of typically developing children (*n* = 24); therefore, their data on typically developing children were used as reference data and were compared to the data of young children from the current study. 

## 2. Materials and Methods

### 2.1. Participant Recruitment and Screening Procedure

This cross-sectional survey study recruited parents who have a child aged younger than 18 years and clinically diagnosed with ASD living in Florida. Through the Center for Autism and Related Disabilities (CARD), other local centers for autism, clinics, and schools, parents were invited to participate in the study from July 2017 until March 2018. A flyer with the research coordinator’s contact information was sent out to parents or posted on social media sites with permission. ASD diagnosis performed by one or more clinical professionals among neurologist, developmental pediatrician, psychiatrist, clinical psychologist, pediatrician, or primary care physician was required to be included in the study. Exclusion criteria included having a serious medical condition that required special treatment or on medication that could affect child’s appetite and body weight such as Olanzapine, Risperidone, or Aripiprazole to avoid potential influences on eating habits or mealtime behaviors. No participant was excluded during the screening process. Sixty parents contacted the research team, and 41 parents completed the survey (68.3% participation rate). A main reason for low responders was a severe hurricane in September 2017; some became overwhelmed by the situation, and some lost interest after a while. The rest did not respond to several follow-up messages without providing reasons.

### 2.2. Demographic Characteristics

Table 1 shows demographic characteristics of the study participants. The majority of the children in our sample (73%) were boys and White (39%) or Hispanic (34%). The majority of parent participants were mother of the child (95%) and had an Associate’s degree or higher (68%). The mean age of the parents was 40.3 years. Breakdowns of demographic characteristics by children’s age group (early childhood = 2–6 years, mid-childhood = 7–11 years, and adolescents = 12–17 years) are also presented in Table 1. There were high percentages of Hispanic participants in the early childhood group (44%) and in the adolescent group (46%), while the majority of participants were White in the mid-childhood group (64 %).

### 2.3. Instrument and Data Collection

Parents completed a 48-question survey via Qualtrics online software. A paper-and-pencil version of an instrument developed by Provost et al. [19] was obtained and transferred verbatim into an online format. The authors reported that face and content validity was conducted by an interdisciplinary panel of experts with extensive experience working with children with ASD, and the instrumentation was pilot tested with a group of families and suggestions were incorporated into the final version [19]. The survey provides primarily nominal data (e.g., yes/no answers, checklists, open-ended questions) on early history, mealtime location, mealtime behaviors, and food preferences and restrictions. The questions were divided into four sections: Early History, Mealtime Environment, Child Likes and Dislikes, and Parental Views.

Three questions on Early History included, “Are you nursing or did you nurse your child?”; “How would you have described your child’s eating behavior in the first three months of life?”; and “Did you have any concerns about your child’s eating when your child was: (a) Birth to 1 year, (b) 1 year to 2 years, (c) 2 years to 3 years, and (d) 3 years to present?”

Ten questions belonged to the Mealtime Environment section. Mealtime location was measured with one question, “Does your child have difficulty eating at: Home, school, fast-food restaurants, regular restaurants, picnics, relatives’ homes, friends’ home, or other places?” Parents were asked to check all response options that apply to them. Examples of other questions in the Mealtime Environment section included types of utensils, whether a child eats independently, who monitors the child’s mealtimes, where the child eats, and how parents know when to feed their children.

Fourteen questions asked child’s preferences and restrictions in the Child Likes and Dislikes section. Questions included food preferences, craving or avoiding certain foods, favorite food textures, temperature, colors, or food packaging, or whether the child smelled food before eating or mouthing/swallowing non-food items. Two questions asked whether the child required food to be prepared a special way and whether the child ate the same food in a repetitive manner every day, which indicate issues with routines or rituals. 

The section on Parental Views included 21 questions on mealtime behaviors, health concerns, food allergies, special diet, and other eating and lifestyle issues and concerns. Mealtime behaviors were asked with one checklist type question with 19 mealtime related problematic behavior response options. 

A total mealtime behavior score, a sum of 44 items from different sections, was calculated [19]. The total score represents overall problems associated with mealtime behavior and food habits, and a higher score indicates more problematic mealtime behaviors. The Cronbach’s alpha value for internal consistency reliability was 0.82 with our sample, suggesting high internal consistency [20].

Potential participants who initially contacted the project coordinator were invited for the survey. Personal survey links were emailed to parents for them to complete the survey online. Follow-up messages were sent periodically if necessary. The consent process was done through the instructional page of the survey. Details of research description, risks and benefits, confidentiality, and how survey results will be used were presented and a downloadable pdf file was available for parent. Parents were informed that they could withdraw from the study at any time. The study protocol was approved by the Institutional Review Board of University of South Florida.

### 2.4. Statistical Analysis

Descriptive statistics were performed for all survey questions, and analyses were performed in two steps. First, subgroup data from children aged 3 to 6 years were compared to the results of Provost et al. [19] to evaluate whether the results from this study differ from the ones in the reference study. Second, data from our whole sample were broken down into early childhood (ages 2–6 years), mid-childhood (ages 7–11 years) and adolescents (ages 12–17 years) to compare results within our sample.

To compare data of children aged 3 to 6 years between our subgroup and the reference, Fisher’s exact test was used for count data and summary independent-samples t-test was performed for the total mealtime behavior score. Frequencies, means, and standard deviations from the original study were extracted and used in the comparison analyses. Chi square tests and Kruskal Wallis tests were used to compare data within our sample by age group. Descriptive statistics and t-tests were analyzed with Statistical Package for the Social Sciences (SPSS) version 24.0 (IBM SPSS, Inc., Chicago, IL, USA). An online analytical software GraphPad QuickCalcs was used for Fischer’s exact test.

## 3. Results

### 3.1. Results by Age Group

#### 3.1.1. Early History

Early history results by age group are presented in Table 2. For all age groups, half or more of parents reported that they nursed their children (50% to 88%) and experienced difficulties breastfeeding (57% to 64%). Fewer early feeding concerns (0–3 years) were reported in mid-childhood and adolescents compared to those in early childhood. However, feeding concerns at ages 3 and older was high for all three age groups (75% in early childhood, 86% in mid-childhood, and 73% in adolescents) with no statistically significant difference.

Having no difficulty eating in any setting and eating a variety of foods were more frequently reported by parents of children aged 7 and older. Only one parent (6%) in the early childhood group reported no difficulty eating in any setting and eating a variety of foods. The percentage of parents who reported that their children have no difficulty eating in any setting increased to 14% in the mid-childhood group and 27% in the adolescent group, while the percent who reported that their children eat a variety of foods increased to 36% in the mid-childhood group and nearly half (46%) in the adolescent group.

#### 3.1.2. Mealtime Environment

Table 3 presents mealtime environment results by age group. While half or more parents (50% to 69%) of children in the early childhood group reported eating difficulties for all seven locations, most parents did not report difficulties at these locations for mid-childhood and adolescents. Regarding eating difficulties, statistically significant differences were detected for eating difficulty at home (*p* = 0.013), at fast food restaurants (*p* = 0.005), and at regular restaurants (*p* = 0.016) by age group, with prevalence decreasing with age for all three variables. Regarding the ways in which parents get their children to eat, there were statistically significant differences for getting children to eat by using positive reinforcements (*p* = 0.02) and for the child eating automatically without assistance (*p* = 0.037), with prevalence decreasing and increasing with age, respectively. 

#### 3.1.3. Child Likes and Dislikes

The results on parent-reported child likes and dislikes indicate that some food preferences were reported nearly in all age groups (88% in the early childhood, and 100% in both mid-childhood and adolescents). Child craving certain foods was reported by 88% of parents in the early childhood group, 79% of parents in the mid-childhood group, and 46% of parents in the adolescent group (*p* = 0.045). The most commonly reported food resistance/restrictions across all age groups were avoiding certain foods (94% in early childhood, 93% in mid-childhood, and 91% in adolescents ) and resisting trying new foods (81% in early childhood, 71% in mid-childhood, and 82% in adolescents ). There was a statistically significant age difference for requiring foods to be prepared in a special way (69% in early childhood, 36% in mid-childhood, and 18% in adolescents; *p* = 0.025), with prevalence decreasing with age. Eating the same food in a repetitive manner (44% in early childhood, 64% in mid-childhood, and 36% in adolescents) was a common feature in all age groups with no statistical difference.

#### 3.1.4. Parental Views

Parental views on mealtime behaviors, issues with routines or rituals, and eating problems decreased with age, while some characteristics were highest in the mid-childhood age group. The most commonly reported mealtime behavior was being a picky eater, which was reported by 75% of parents of children in the early childhood group, 71% in the mid-childhood group, and 46% in the adolescent group. The second most commonly reported behavior was leaving the table frequently, which was reported by 71% of the mid-childhood group, 50% of the early childhood group, and 27% of the adolescent group. Stuffing mouth or cheeks was also a commonly reported eating problem, which was reported by 50% of parents in the early childhood group, 21% in the mid-childhood group, and 27% in the adolescent group. 

Statistically significant differences by age group were detected for gagging (31% in early childhood, 7% in mid-childhood, and 0% in adolescents; *p* = 0.049), child playing with his/her food (50% in early childhood, 29% in mid-childhood, and 0% in adolescents; *p* = 0.019), and eating very slowly (56% in early childhood, 71% in mid-childhood, and 18% in adolescents; *p* = 0.027). In addition, there was a statistically significant difference for whether parents reported mealtime with their children to be stressful, O.K., or fun (*p* = 0.036), with the majority of parents (56%) in the early childhood group reporting mealtime to be stressful, and the majority of parents in the mid-childhood and adolescent groups (64% for each group) reporting mealtime to be O.K. Not surprisingly, the child was reported to be toilet trained for urine (*p* = 0.018), with prevalence increasing with age.

Parents reported a variety of health concerns and special diets, and there was no statistically significant difference by age group. The most commonly reported health concern that the child experienced in the past was an ear infection (31% in early childhood, 43% in mid-childhood, and 64% in adolescents), while the most frequently reported health concerns that parents currently have was having to take medications (13% in early childhood, 50% in mid-childhood, and 27% in adolescents). Regarding special diets, parents reported that they currently apply avoiding food additives/color (31% in early childhood, 14% in mid-childhood, and 27% in adolescents), gluten free diet (6% in early childhood, 14% in mid-childhood, and 9% in adolescents), lactose free diet (19% in early childhood, 21% in mid-childhood, and 9% in adolescents), or casein free diet (13% in early childhood, 14% in mid-childhood, and 9% in adolescents). Some parents reported that they tried these various special diets in the past, on average for about 2 to 5 months, for their children. Notably, parents in the adolescent group reported that they tried lactose-free diet (36%) and/or casein-free diet (36%) in the past, and among those, one parent reported that the child is still on both lactose-free and casein-free diet. 

#### 3.1.5. Total Score for Mealtime Behavior Scale

The total mealtime behavior score ranged from 6 to 22 with a mean of 15.88 (SD = 4.96) for the early childhood group, from 3 to 24 with a mean of 13.57 (SD = 6.36) for the mid-childhood group, and from 2 to 15 with a mean of 7.91 (SD = 4.41) for the adolescent group. The mean scores for the early childhood (*p* < 0.001) and mid-childhood groups (*p* = 0.005) were significantly higher compared to the one in the adolescent group, indicating significantly more problematic mealtime behaviors.

### 3.2. Replication Study: Comparisons between a Sample of 3–6 Years Old Children with ASD from the Current Study and the Reference Data from Provost et al. [19]

The mean age of the sample of 3–6 years old children with ASD in the current study was 4.4 years (SD = 1.06), similar to the ones (4.3 years both in the ASD and the typically developing groups) in the Provost et al. [19]. The percentage of boys in the current study (73%) was also similar to the ones in the reference study (75% both in the ASD and the typically developing children). The compositions of race/ethnicity were slightly different between the current study and the reference study. The percentages of Hispanic or Latino were similar (40% in the current study vs. 46% in the reference study). However, there was a higher percentage of other race/ethnicity in the current study (27% in the current study vs. 4% in the reference study), while there was a higher percentage of White in the reference study (27% in the current study vs. 50% in the reference study).

#### 3.2.1. Early History

Table 4 presents results regarding early history compared to the reference data. In our sample, 13 out of 15 mothers nursed their children, and 69% reported difficulties with breastfeeding, which was significantly greater from the 15% of mothers who reported difficulties with breastfeeding in typically developing children. A significantly lower percentage of participants in our sample reported strong continuous sucking behavior compared to the reference data for both ASD and typically developing children. There were significantly more parents in our sample who reported feeding concerns when their children were 1–2 years, 2–3 years, and from 3 years of age until present compared to those of typically developing children in the reference sample. Significantly fewer parents of children with ASD in our sample reported that their children have no difficulty eating in any setting (7%) and eat a variety of foods (7%) compared to the typically developing reference data (50% and 58%, respectively). 

#### 3.2.2. Mealtime Location

Results regarding mealtime locations are presented in Table 5. Compared to the reference data, significantly more parents of children in our sample reported that their children have eating difficulties at home (47%) and at fast food restaurants (67%) than both ASD (13% and 21%) and typically developing children (13% and 17%) from the reference data, respectively. In addition, 47% of parents in our sample reported that their children have eating difficulties at school, while no parents of typically developing children from the reference sample reported eating difficulties at school (*p* < 0.001). Eight parents (53%) from our sample reported that their children have eating difficulties at picnics, which was significantly different from the typically developing children reference data (17%; *p* < 0.05).

#### 3.2.3. Parental Views on Mealtime Behaviors, Issues with Routines or Rituals and Eating Problems

Table 6 shows parental views on mealtime behaviors, issues with routines and rituals, and various eating problems. Most participants in our sample reported that their children leave the table frequently at mealtime (51%) and are picky eaters (66%). There were 39% who reported their children to be restless but sit during meals, 32% as inconsistent eaters, and 22% as ritualistic eaters. Significantly more parents of children with ASD in our sample identified their children as picky eaters (73% vs. 25%) and as having problems with gagging while eating (33% vs. 0%), compared to the reference sample of typically developing children (all *p* < 0.01). Additionally, significantly more parents in our sample reported that their child requires food prepared in a special way (67% vs. 25%), eats the same food in a repetitive manner (47% vs. 13%) and stuffs mouth or cheeks during eating (53% vs. 13%) compared to the reference sample of typically developing children (all *p* < 0.05). A significantly smaller number of parents reported their child as a ritualistic eater in our sample compared to the reference sample of children with ASD (20% vs. 33%; *p* < 0.05). 

#### 3.2.4. Food Preferences and Restrictions

Table 7 reports results of food preferences, food resistance/restrictions, and smelling/mouthing of food items. Significantly more parents of children in our sample reported that their children crave certain foods (93% vs. 54%) and mouth non-food items (46% vs. 13%) compared to the typically developing reference group (both *p* < 0.05). Significantly more parents of children with ASD in our sample (60%) indicated that their children have favorite food textures compared to the reference sample of typically developing children (4%; *p* < 0.001). Other food preferences and restrictions were not significantly different from the reference data. 

#### 3.2.5. Total Score for Mealtime Behavior Scale

The total mealtime behavior score was constructed from 44 items included in Table 2, Table 3, Table 4 and Table 5 and can range from 0 to 44, with a higher score indicating more problems associated with mealtime behavior and food habits. A significant difference was found in mean total score between children with ASD aged 3–6 years in this study (mean = 15.93, SD = 5.13) and typically developing children from Provost et al. (mean = 6.3, SD = 4.3; *p* < 0.001). The higher mean mealtime behavior score in children with ASD suggests significantly more problematic behaviors compared to typically developing children. There was no significant difference between children with ASD from this study and those with ASD from the reference data.

## 4. Discussion

In this study, a survey tool [19] was used to examine early history, mealtime environment, food preferences, and parental views on mealtime and eating behaviors among children with ASD in Florida. Parents who had children aged 2–17 years with ASD completed the survey online between July 2017 and March 2018. 

The results of this study show that the percentages of parents who reported problematic mealtime behaviors and concerns were generally high for children of all ages, indicating that parental concerns regarding children’s mealtime and eating behaviors persist throughout childhood and adolescent years. The analysis of our sample by age group suggested that picky eating may diminish over time in children with ASD. It is also possible that parents may repeatedly feed their children with their favorite foods and children eat more independently as they grow older, and therefore parents’ perceived picky eating behaviors of their children may diminish even if the actual food intake may not change. The number of parents who reported that their children have no difficulty eating in any setting and eat a variety of foods increased from 6% to 36% and 46% from the early childhood age group to the mid-childhood and the adolescent age group, respectively. Many statistically significant differences indicated the highest prevalence of eating difficulties, problematic mealtime behaviors, and other issues in the early childhood group and a decreasing prevalence with age. The total mealtime behavior scale scores reiterate the higher prevalence of problems associated with mealtime behavior and food habits in younger children, as total scores were significantly higher in children ages 2–6 compared to children ages 7–11 and ages 12–17. However, it should be noted that recall bias may have impacted responses from parents of older children who were asked to recall information from several years ago. Age specific interventions may be beneficial for the eating difficulties and problematic mealtime behaviors that are specifically more prevalent in younger children with ASD. 

While many reported problematic behaviors seemed to be age-dependent, others were consistently high among all three age groups. For example, prevalence of feeding concerns at ages 3 and older, exhibiting specific food preferences, and avoiding certain foods were all high for all three age groups. These findings suggest that certain problematic behaviors start early and persist until ages as old as 17 years in children with ASD. On the other hand, some (e.g., eating the same food in a repetitive manner, leaving the table frequently) were highest in the mid-childhood group, which may indicate that some habits and behaviors develop later in mid-childhood. Information on a subsample—children aged 3–6 years with ASD—was compared to information from the Provost et al. study [19]. Data from both children with ASD and typically developing children from the reference were compared to see whether our sample data was different from the reference data with and without ASD. Children aged 3–6 years with ASD in the current study showed surprising similar outcomes in most areas to those with ASD in the reference group from Provost et al. [19]. There was a significant difference in one specific early eating behavior: A lower percentage of parents in our sample reported that their children had easy feeding with strong continuous sucking in the first 3 months after birth. In addition, significantly higher percentages of participants in the current study reported that their children had eating difficulties at home and at fast food restaurants. This might be due to the different study locations and slightly different demographic characteristics between the two studies. However, further research is required to determine whether eating difficulties at home and at fast food restaurants are influenced by region. Compared with typically developing children from the reference study, significantly more parents in our study reported that their children had eating difficulties at school, regular restaurants, and picnics, indicating that many children with ASD have mealtime issues when eating out, regardless of location. The majority of previous studies examining problematic mealtime behaviors in children with ASD do not have any information on specific mealtime locations [2]. Possible reasons for this finding are that locations outside of the home impact parent’s ability to effectively manage the behavior, or the change in routine and additional social factors outside of home mealtimes intensify problematic behavior. This might also increase parental stress and affect their quality of life in general. Food selectivity and problematic mealtime behaviors are often addressed only in clinical settings or for parents to intervene, mostly at home. Clearly, parents of children with ASD would benefit from intervention approaches that allow for community-based practice. For example, early intervention providers within the birth-3 Part C Individuals with Disabilities Education Act (IDEA) program are required to provide intervention services in the natural environment [21]. As part of this natural environment, the interventionist may decide to accompany the family to a restaurant or park picnic table to practice age-appropriate mealtime behaviors and support parents in implementing effective behavioral feeding strategies (e.g., positive reinforcement [22]). Further efforts to increase public awareness about feeding and mealtime behavioral challenges among families with ASD might be beneficial. Having restaurants and other facilities with an atmosphere that is more comfortable for children with special needs and their families might also be helpful. 

The findings from this study indicate that atypical eating behaviors in infancy may be noticeable as early as in the first 3 months. A significantly higher number of parents in our study reported that they had concerns about their child’s eating at 1–2 years of age, compared to parents of typically developing children in the reference study, which is consistent with the results of Provost et al.’s study. More than 70% of parents of 3- to 6-year-old children with ASD in both our study and the reference study reported that they had feeding concerns when their children were 2–3 years old, while only 17% of parents with typically developing children reported concerns. Of the limited research on early history of feeding difficulties, parents reported signs of problematic eating/mealtime behaviors by 6 months of age [23]; however, more data is needed to determine a proper age to start screening various feeding issues. Understanding early history and atypical eating behavioral patterns may help with early diagnosis of the condition and the design of intervention techniques to improve eating habits. The birth-3 Part C program is well-positioned to screen infants and toddlers for such feeding difficulties and to develop and implement early intervention programs. Further evidence is needed to conclude whether children having early feeding difficulties before their first birthday is associated with ASD and other developmental disabilities. 

The overall mealtime behavior scale indicated that our sample of children with ASD had significantly more problematic behaviors than the typically developing children in the reference study. Item-by-item analyses show several specific differences between children aged 3–6 years in our sample and typically developing children. Those behaviors were mostly related to food preference or rigidity rather than behavioral issues such as tantrums or being restless at mealtime, which were also common in typically developing children. A significantly greater number of children with ASD in our sample were considered picky eaters, require food prepared in a special way, had a problem with gagging, craved certain foods, and had favorite food textures. These features have been frequently reported as problematic among children with ASD [24,25]. Consistent with other studies [2,10,25,26], the parents in our study also reported that their children did not eat a variety of foods: Only one out of 15 children aged 3 to 6 years old in our sample (7%) ate a variety of foods, while almost 60% of typically developing children in the reference study did so.

Sensory difficulties along with behavioral rigidity and impaired social learning could likely contribute to the differences found between children with ASD and typically developing children [27]. Several studies have shown that significant sensory differences may exist between children with ASD and typically developing children, which can cause the evidenced difficulties with eating, e.g., selective eating or food refusal [28,29,30,31,32]. However, it is still unclear whether sensory-based therapies will improve mealtime behavior and selective eating problems [33]; therefore, further intervention studies examining efficacy of such strategies are warranted and large-scale research on the most effective strategies to address these difficulties (whether sensory based or behavioral) are needed. 

The limitations of the current study include that we used a convenience sample and a parent-report method of data collection. It is possible that the parents who participated in this study may have had more severe mealtime behavior or nutritional concerns regarding their children. Additionally, parents were asked to recall details about their children’s early feeding history. Parents of older children in particular may have had a difficult time recalling such information. In addition to the recall bias limitation, our sample has a limitation of small and unequal group sizes, with the 12–17 age group having only 11 children. More research is needed to examine further age differences in mealtime behaviors and eating habits among children and adolescents with ASD. Another limitation is that we did not collect our own control group with parents of typically developing children, but using the typically developing control from the study that used exactly the same questionnaire was beneficial. Further studies in the same regions with similar demographic characteristics would provide better matched comparisons. The results of this study are not generalizable to other settings and populations with different demographic backgrounds. Lastly, even though the instrument was previously tested for face and content validity [19] and the internal consistency reliability was high in the current study, to utilize this instrument more broadly, further studies assessing its construct validity and reliability are necessary. Despite these limitations, this is one of a few studies to measure early history of feeding difficulties and feeding environment among children with ASD. In addition, since the study participants were from across Florida, the findings of this study may inform professionals and agencies that work with children on the spectrum and their families, particularly in Florida. This is also one of few studies that included a wide range of age groups and compared outcomes by three age categories (i.e., early childhood, mid-childhood, and adolescents), making it possible to see whether common mealtime and eating problems persist until adolescence and whether there are significant differences among age groups. 

As there are growing health concerns such as obesity in populations with ASD, more research is needed to investigate how the mealtime and eating behavior problems are associated with health outcomes such as obesity or diabetes, and what intervention strategies can effectively address both problematic mealtime behaviors and the health outcomes in children with ASD. In addition, it would be worth further investigating whether mealtime behaviors are directly associated with potential nutrient deficiencies. 

## 5. Conclusions

The results of this study confirm previous findings that children with ASD exhibit significantly more problematic mealtime behaviors than typically developing children. Some of these problems, such as eating difficulties in various settings, may diminish with age, while others, such as avoiding certain foods, may persist through adolescence. Features of feeding issues identified in this study included difficulties in early feeding (i.e., breastfeeding), not eating a variety of foods, difficulties of eating at different locations, being a picky eater, ritualistic manners, gagging, stuffing mouth or cheeks with foods, craving certain foods, having favorite food textures, and mouthing nonfood items. Our findings with regard to age group suggest that early childhood intervention for feeding difficulties in children with ASD are warranted. Further research in adolescents with ASD and whether problematic mealtime behaviors are associated with nutritional inadequacy or other health outcomes is needed. Lastly, it might be worth exploring whether an early screening for feeding difficulties can be used as a potential criterion for ASD diagnosis.

## Figures and Tables

**Table 1:** Demographic characteristics of study participants.

Variables	Total Sample 2–17 Years (*n* = 41)	Early Childhood 2–6 Years (*n* = 16)	Mid-Childhood 7–11 Years (*n* = 14)	Adolescents 12–17 Years (*n* = 11)
Child with ASD demographics				
Age (years) ^a^	8.3 ± 4.02	4.25 ± 1.18	8.64 ± 1.55	13.55 ± 1.86
Gender				
Boys	30 (73%)	12 (75%)	11 (79%)	7 (64%)
Race/ethnicity				
White	16 (39%)	4 (25%)	9 (64%)	3 (27%)
Black	3 (7%)	0 (0%)	1 (7%)	2 (18%)
Hispanic or Latino	14 (34%)	7 (44%)	2 (14%)	5 (46%)
Asian	1 (2%)	1 (6%)	0 (0%)	0 (0%)
Other	7 (17%)	4 (25%)	2 (14%)	1 (9%)
Parent demographics				
Mother	39 (95%)	15 (94%)	14 (100%)	10 (91%)
Age (years) ^a^	40.3 ± 7.7	35.44 ± 5.61	40.92 ± 5.14	47.40 ± 8.11
Race/ethnicity				
White	20 (49%)	7 (44%)	10 (71%)	3 (27%)
Black	4 (10%)	1 (6%)	1 (7%)	2 (18%)
Hispanic	14 (34%)	7 (44%)	2 (14%)	5 (46%)
Asia	1 (2%)	1 (6%)	0 (0%)	0 (0%)
Other	2 (5%)	0 (0%)	1 (7%)	1 (9%)
Highest level of education				
High school diploma or equivalent	1 (2%)	0 (0%)	1 (7%)	0 (0%)
Some college, no degree	12 (29%)	7 (44%)	2 (14%)	3 (27%)
Associate’s degree	5 (12%)	2 (13%)	1 (7%)	2 (18%)
University or 4-year degree	11 (27%)	3 (19%)	6 (43%)	2 (18%)
Post-college or graduate degree	12 (29%)	4 (25%)	4 (29%)	4 (36%)

^a^ Mean ± standard deviation. NA represents not available.

**Table 2:** Early history by age group.

Item	Early Childhood 2–6 Years (*n* = 16)	Mid-Childhood 7–11 Years (*n* = 14)	Adolescents 12–17 Years (*n* = 11)	Chi-Square Test, *p*-Value
Nursing				
Nurse your child	14 (88%)	7 (50%)	7 (64%)	0.082
Difficulties with breastfeeding ^a^	9 (64%)	4 (57%)	4 (57%)	0.810
Eating behavior in first 3 months (bottle or breastfeeding)				
Strong continuous suck taking 10–15 min. Easy to feed.	4 (25%)	6 (43%)	5 (46%)	0.464
Strong continuous suck but frequent breaks requiring to redirect	2 (13%)	3 (21%)	2 (18%)	0.805
Unorganized sucking behavior requiring your assistance Slow eater	5 (31%)	6 (43%)	1 (9%)	0.179
Irritable eater, fidgety, frequent release of nipple with crying	1 (6%)	2 (14%)	1 (9%)	0.758
Required frequent feedings	4 (25%)	4 (29%)	3 (27%)	0.975
Gagging, choking, facial grimacing and spitting up. Difficult to feed	3 (19%)	2 (14%)	0 (0%)	0.328
Concerns about eating				
Feeding concerns, birth to 1 year	7 (44%)	11 (79%)	6 (55%)	0.147
Feeding concerns, 1–2 years	10 (63%)	8 (57%)	5 (46%)	0.678
Feeding concerns, 2–3 years	14 (88%)	8 (57%)	5 (46%)	0.054
Feeding concerns, 3 years to present	12 (75%)	12 (86%)	8 (73%)	0.688

^a^ Percentage is derived from total participants who nursed their child.

**Table 3:** Mealtime environment by age group.

Item	Early Childhood 2–6 Years (*n* = 16)	Mid-Childhood 7–11 Years (*n* = 14)	Adolescents 12–17 Years (*n* = 11)	*p*-Value
Utensils child currently uses for eating independently				
Bottle	4 (25%)	1 (7%)	0 (0%)	0.116
Straw	9 (56%)	10 (71%)	8 (73%)	0.582
Cup	11 (69%)	9 (64%)	9 (82%)	0.617
Fingers	14 (88%)	12 (86%)	6 (55%)	0.088
Spoon	13 (81%)	9 (64%)	8 (73%)	0.578
Fork	12 (75%)	8 (57%)	10 (91%)	0.163
Other utensils	3 (19%)	3 (21%)	5 (46%)	0.261
Child eats independently except for needing his or her food to be cut up	14 (88%)	12 (86%)	11 (100%)	0.438
Where does child eat				
In high chair	3 (19%)	0 (0%)	0 (0%)	0.080
On feeder’s lap	0 (0%)	0 (0%)	0 (0%)	N/A
On chair by self	12 (75%)	12 (86%)	8 (73%)	0.688
On chair with family	2 (13%)	5 (36%)	6 (55%)	0.065
Other	4 (25%)	3 (21%)	0 (0%)	0.206
Approximately how long does it take for the child to eat in minutes ^a^				
Breakfast	22 ± 15.11	15.42 ± 16.94	12.40 ± 5.04	0.228
Lunch	29.33 ± 18.31	29.17 ± 20.21	15.00 ± 4.33	0.115
Dinner	32.47 ± 19.84	30.58 ± 22.45	21.11 ± 9.61	0.356
Snacks	13.67 ± 8.37	14.11 ± 12.93	10.20 ± 6.78	0.592
Besides breakfast, lunch and dinner, how many times during the day does the child eat snacks ^a^	2.25 ± 0.68	2.36 ± 0.93	2.45 ± 1.04	0.972
Mealtime Location				
Eating difficulty at home	8 (50%)	3 (21%)	0 (0%)	0.013 *
Eating difficulty at school	8 (50%)	5 (36%)	3 (27%)	0.469
Eating difficulty at fast food restaurants	11 (69%)	4 (29%)	1 (9%)	0.005 **
Eating difficulty at regular restaurants	11 (69%)	4 (29%)	2 (18%)	0.016 *
Eating difficulty at picnics	9 (56%)	4 (29%)	2 (18%)	0.097
Eating difficulty at relatives’ homes	10 (63%)	6 (43%)	2 (18%)	0.074
Eating difficulty at friends’ homes	8 (50%)	7 (50%)	3 (27%)	0.430
Other places	4 (25%)	5 (36%)	5 (46%)	0.539
How do parents know when to feed their child				
On demand	10 (63%)	10 (71%)	5 (46%)	0.412
At specific mealtimes	12 (75%)	11 (79%)	6 (55%)	0.377
When he/she appears hungry	6 (38%)	4 (29%)	2 (18%)	0.554
When parents think he/she should be hungry	7 (44%)	5 (36%)	2 (18%)	0.383
Other	2 (13%)	3 (21%)	4 (36%)	0.338
How do parents get their child to eat				
Coax	6 (38%)	7 (50%)	2 (18%)	0.260
Time out for not eating	1 (6%)	1 (7%)	0 (0%)	0.676
Ignore non-eating behaviors	1 (6%)	2 (14%)	0 (0%)	0.387
Put food in the child’s mouth	6 (38%)	3 (21%)	1 (9%)	0.228
Use hand over hand assistance to put food in mouth	4 (25%)	1 (7%)	1 (9%)	0.321
Positive reinforcements	10 (63%)	10 (71%)	2 (18%)	0.020 *
Pretend to eat and enjoy food	4 (25%)	2 (14%)	1 (9%)	0.527
Get angry: make child finish food	2 (13%)	3 (21%)	0 (0%)	0.267
Games (airplane in the hangar)	3 (19%)	0 (0%)	0 (0%)	0.080
Child eats automatically	7 (44%)	7 (50%)	10 (91%)	0.037 *
Other	5 (31%)	4 (29%)	3 (27%)	0.973
How do parents know that the child is finished eating				
Child gives gesture	1 (6%)	2 (14%)	1 (9%)	0.758
Child turns face away	3 (19%)	2 (14%)	1 (9%)	0.783
Child stops opening mouth	2 (13%)	0 (0%)	0 (0%)	0.193
Child says “I am done”	10 (63%)	9 (64%)	6 (55%)	0.873
Child throws food	1 (6%)	0 (0%)	0 (0%)	0.449
Other	8 (50%)	8 (57%)	6 (55%)	0.924

Bold type indicates significant results; Significance at: * *p* < 0.05, ** *p* < 0.01; ^a^ Mean ± standard deviation; Chi square test used for categorical variables and Kruskal Wallis test used for continuous variables.

**Table 4:** Early history of feeding and eating behaviors.

	Current Study	Reference Study ^a^	
Item	ASD 3–6 Years (*n* = 15)	ASD 3–6 Years (*n* = 24)	Typically Developing Children 3–6 Years (*n* = 24)
Nursing			
Nurse your child	13 (87%)	18 (75%)	20 (83%)
Difficulties with breastfeeding ^b^	9 (69%)	7 (39%)	3 (15%) *
Eating behavior in first 3 months (bottle or breastfeeding)			
Strong continuous suck taking 10–15 min. Easy to feed	4 (27%)	15 (63%) *	19 (79%) **
Strong continuous suck but frequent breaks requiring to redirect	2 (13%)	2 (8%)	2 (8%)
Unorganized sucking behavior requiring your assistance. Slow eater	5 (33%)	5 (21%)	1 (4%) *
Irritable eater, fidgety, frequent release of nipple with crying	1 (7%)	4 (17%)	0 (0%)
Required frequent feedings	4 (27%)	11 (46%)	7 (30%)
Gagging, choking, facial grimacing and spitting up. Difficult to feed	2 (13%)	5 (21%)	3 (13%)
Concerns about eating			
Feeding concerns, birth to 1 year	7 (47%)	9 (38%)	7 (30%)
Feeding concerns, 1–2 years	10 (67%)	12 (50%)	2 (8%) ***
Feeding concerns, 2–3 years	14 (93%)	17 (71%)	4 (17%) ***
Feeding concerns, 3 years to present	12 (80%)	16 (67%)	4 (17%) ***
Current overview			
Has no difficulty eating in any setting	1 (7%)	5 (21%)	12 (50%) **
Eats a variety of foods	1 (7%)	3 (13%)	14 (58%) **

^a^ Provost et al. (2010) [19]; ^b^ Percentage is derived from total participants who nursed their child; Bold type indicates significant results; Fisher’s exact test significance at: * *p* < 0.05, ** *p* < 0.01, *** *p* < 0.001.

**Table 5:** Eating difficulties at mealtime locations.

	Current Study	Reference Study ^a^	
Item	ASD 3–6 Years (*n* = 15)	ASD 3–6 Years (*n* = 24)	Typically Developing Children 3–6 Years (*n* = 24)
Mealtime Location			
Eats only in specific places	1 (7%)	5 (21%)	1 (4%)
Eating difficulty at home	7 (47%)	3 (13%) *	3 (13%) *
Eating difficulty at school	7 (47%)	6 (25%)	0 (0%) ***
Eating difficulty at fast food restaurants	10 (67%)	5 (21%) **	4 (17%) **
Eating difficulty at regular restaurants	10 (67%)	13 (54%)	2 (8%) ***
Eating difficulty at picnics	8 (53%)	9 (38%)	4 (17%) *
Eating difficulty at relatives’ homes	9 (60%)	11 (46%)	7 (29%)
Eating difficulty at friends’ homes	7 (47%)	12 (50%)	7 (29%)

^a^ Provost et al. (2010) [19]; Bold type indicates significant results; Fisher’s exact test significance at: * *p* < 0.05, ** *p* < 0.01, *** *p* < 0.001.

**Table 6:** Parental views on mealtime behaviors, issues with routines or rituals, and eating problems.

	Current Study	Reference Study ^a^	
Item	ASD 3–6 Years (*n* = 15)	ASD 3–6 Years (*n* = 24)	Typically Developing Children 3–6 Years (*n* = 24)
Mealtime behaviors: My child,			
Leaves table frequently	8 (53%)	14 (58%)	7 (29%)
Restless but sits	9 (60%)	12 (50%)	11 (46%)
Resists sitting at table	3 (20%)	11 (46%)	3 (13%)
Seems fearful sitting at	0 (0%)	0 (0%)	1 (4%)
Rocks during meals	1 (7%)	1 (4%)	0 (0%)
Hits self while eating	0 (0%)	1 (4%)	0 (0%)
Has frequent tantrums	1 (7%)	6 (25%)	0 (0%)
Uses only specific utensils	2 (13%)	4 (17%)	0 (0%)
Throws/dumps food	1 (7%)	8 (33%)	1 (4%)
Spits food	0 (0%)	5 (21%)	1 (4%)
Is a picky eater	11 (73%)	18 (75%)	6 (25%) **
Is an inconsistent eater	7 (47%)	10 (42%)	10 (42%)
Issues with routines or rituals: My child,			
Is a ritualistic eater	3 (20%)	8 (33%)	2 (8%)
Requires food prepared in a special way	10 (67%)	12 (50%)	6 (25%) *
Eats same food in a repetitive manner	7 (47%)	10 (42%)	3 (13%) *
Has routines or rituals with food or eating	2 (13%)	9 (38%)	4 (17%)
Is upset if routine is broken	3 (20%)	6 (25%)	3 (13%)
Needs food in specific place on plate	1 (7%)	3 (13%)	0 (0%)
My child has problems with			
Gagging	5 (33%)	8 (33%)	0 (0%) **
Chewing	0 (0%)	0 (0%)	0 (0%)
Swallowing	0 (0%)	5 (21%)	0 (0%)
Sucking	0 (0%)	0 (0%)	0 (0%)
Throwing up	0 (0%)	0 (0%)	0 (0%)
Nausea	1 (7%)	2 (8%)	0 (0%)
Stuffing mouth or cheeks	8 (53%)	8 (33%)	3 (13%) *

^a^ Provost et al. (2010) [19]; Bold type indicates significant results; Fisher’s exact test significance at: * *p* < 0.05, ** *p* < 0.01.

**Table 7:** Food preferences and restrictions.

	Current Study	Reference Study ^a^	
Item	ASD 3–6 Years (*n* = 15)	ASD 3–6 Years (*n* = 24)	Typically Developing Children 3–6 years (*n* = 24)
Food craving and preferences			
Child craves certain foods	14 (93%)	20 (83%)	13 (54%) *
Has food preferences	14 (93%)	23 (96%)	21 (88%)
Has favorite food textures	9 (60%)	17 (71%)	1 (4%) ***
Has favorite food colors	1 (7%)	8 (33%)	2 (8%)
Prefers certain food temperature	9 (60%)	11 (46%)	12 (50%)
Food resistance/restrictions			
Child avoids certain foods	14 (93%)	22 (92%)	16 (67%)
Resists trying new food	12 (80%)	22 (92%)	12 (50%)
Limits food to favorite foods	4 (27%)	10 (42%)	5 (21%)
Limits food to favorite texture	3 (20%)	9 (38%)	1 (4%)
Limits food to favorite colors	0 (0%)	1 (4%)	1 (4%)
Limits food to preferred temperature	4 (27%)	5 (21%)	4 (17%)
Limits food to preferred packaging	2 (13%)	2 (8%)	0 (0%)
Smelling/mouthing			
Smells food before eating	6 (40%)	5 (21%)	6 (25%)
Mouths nonfood items	7 (47%)	13 (54%)	3 (13%) *
Swallows nonfood items	3 (20%)	6 (25%)	1 (4%)

^a^ Provost et al. (2010) [19]; Bold type indicates significant results; Fisher’s exact test significance at: * *p* < 0.05, *** *p* < 0.001.
